# Machine Learning Approach to Predict Positive Screening of Methicillin-Resistant *Staphylococcus aureus* During Mechanical Ventilation Using Synthetic Dataset From MIMIC-IV Database

**DOI:** 10.3389/fmed.2021.694520

**Published:** 2021-11-16

**Authors:** Yohei Hirano, Keito Shinmoto, Yohei Okada, Kazuhiro Suga, Jeffrey Bombard, Shogo Murahata, Manoj Shrestha, Patrick Ocheja, Aiko Tanaka

**Affiliations:** ^1^Department of Emergency and Critical Care Medicine, Juntendo University Urayasu Hospital, Chiba, Japan; ^2^Department of Internal Medicine, Tokyo bay Ichikawa Urayasu Medical Center, Chiba, Japan; ^3^Department of Primary Care and Emergency Medicine, Graduate School of Medicine, Kyoto University, Kyoto, Japan; ^4^Department of Mechanical Engineering, Faculty of Engineering, Kogakuin University, Tokyo, Japan; ^5^Dowell Co., Ltd., Hokkaido, Japan; ^6^DeerWalk Japan, Tokyo, Japan; ^7^Graduate School of Informatics, Kyoto University, Kyoto, Japan; ^8^Department of Anesthesiology and Intensive Care Medicine, Osaka University Graduate School of Medicine, Osaka, Japan

**Keywords:** prediction, machine learning, mechanical ventilation, Methicillin-Resistant *Staphylococcus aureus*—MRSA, outcome

## Abstract

**Background:** Mechanically ventilated patients are susceptible to nosocomial infections such as ventilator-associated pneumonia. To treat ventilated patients with suspected infection, clinicians select appropriate antibiotics. However, decision-making regarding the use of antibiotics for methicillin-resistant *Staphylococcus aureus* (MRSA) is challenging, because of the lack of evidence-supported criteria. This study aims to derive a machine learning model to predict MRSA as a possible pathogen responsible for infection in mechanically ventilated patients.

**Methods:** Data were collected from the Medical Information Mart for Intensive Care (MIMIC)-IV database (an openly available database of patients treated at the Beth Israel Deaconess Medical Center in the period 2008–2019). Of 26,409 mechanically ventilated patients, 809 were screened for MRSA during the mechanical ventilation period and included in the study. The outcome was positivity to MRSA on screening, which was highly imbalanced in the dataset, with 93.9% positive outcomes. Therefore, after dividing the dataset into a training set (*n* = 566) and a test set (*n* = 243) for validation by stratified random sampling with a 7:3 allocation ratio, synthetic datasets with 50% positive outcomes were created by synthetic minority over-sampling for both sets individually (synthetic training set: *n* = 1,064; synthetic test set: *n* = 456). Using these synthetic datasets, we trained and validated an XGBoost machine learning model using 28 predictor variables for outcome prediction. Model performance was evaluated by area under the receiver operating characteristic (AUROC), sensitivity, specificity, and other statistical measurements. Feature importance was computed by the Gini method.

**Results:** In validation, the XGBoost model demonstrated reliable outcome prediction with an AUROC value of 0.89 [95% confidence interval (CI): 0.83–0.95]. The model showed a high sensitivity of 0.98 [CI: 0.95–0.99], but a low specificity of 0.47 [CI: 0.41–0.54] and a positive predictive value of 0.65 [CI: 0.62–0.68]. Important predictor variables included admission from the emergency department, insertion of arterial lines, prior quinolone use, hemodialysis, and admission to a surgical intensive care unit.

**Conclusions:** We were able to develop an effective machine learning model to predict positive MRSA screening during mechanical ventilation using synthetic datasets, thus encouraging further research to develop a clinically relevant machine learning model for antibiotics stewardship.

## Introduction

Selection of antibiotics for critically-ill patients undergoing mechanical ventilation in the intensive care unit (ICU) is challenging (1, 2), as these patients are susceptible to nosocomial infections such as ventilator-associated pneumonia (VAP), catheter-related blood site infection, and catheter-associated urinary tract infection (3–5). Thus, multiple anti-bacterial agents with broad spectrum are often empirically selected for the treatment of this population. However, the inappropriate use of broad-spectrum antibiotics could lead to the emergence of resistant bacteria (6, 7). The incorrect usage of antibiotics might also cause adverse effects outweighing their benefits (8). Therefore, optimized antibiotics selection would be beneficial for patient outcomes.

In particular, the decision-making regarding the use of antibiotics for methicillin-resistant *staphylococcus aureus* (MRSA) is a source of distress for clinicians, due to their harmful complications such as hypersensitivity reactions, neutropenia, thrombocytopenia, and acute kidney injury (9–11). Although a variety of risk factors for MRSA colonization have been identified and reported (12, 13), there are currently no specific criteria for the use of antibiotics for MRSA.

To identify patients carrying MRSA, a specific screening test is often used. MRSA detection could be helpful for clinicians not only to determine the choice of antibiotics, but also to identify the patients who could potentially spread MRSA to other patients. However, the commonly used culture screening method for MRSA requires several days to obtain the result, and thus cannot be used to obtain information in real time (14). Hence, the accurate and timely prediction of the presence of MRSA in mechanically ventilated patients would have great significance and impact in the clinical setting.

Recently, machine learning methods have demonstrated their usefulness for clinical decision support in infectious diseases (15). This study aimed to develop and validate a machine learning-based model to predict the presence of MRSA in mechanically ventilated patients by using only available patient data obtained before MRSA screening.

## Materials and Methods

### Data Sources and Ethical Approval

The data for the current retrospective study were obtained from the Medical Information Mart for Intensive Care (MIMIC)-IV database, version 1.4. This publicly available relational database is provided by the Laboratory for Computational Physiology at the Massachusetts Institute of Technology (MIT, Cambridge, MA, USA), and includes information on critical care patients who were admitted to the ICU at the Beth Israel Deaconess Medical Center (BIDMC, Boston, MA, USA) during the period 2008–2019. Patient identifiers were removed according to the Health Insurance Portability and Accountability Act (HIPAA) Safe Harbor provision. Details of the MIMIC-IV database have been described elsewhere (16, 17). The MIMIC-IV project was approved by the Institutional Review Boards of BIDMC and MIT. Requirement for individual patient consent was waived because the project did not impact clinical care and all protected health information was deidentified. Data were extracted by Yohei Hirano, MD, who completed the requested online training course of the Collaborative Institutional Training Initiative (CITI) program (record ID: 38943363) and was approved as credentialed user to access the MIMIC-IV database. The current study was conducted in accordance with the Declaration of Helsinki.

### Study Population and Outcomes

**Figure 1:**
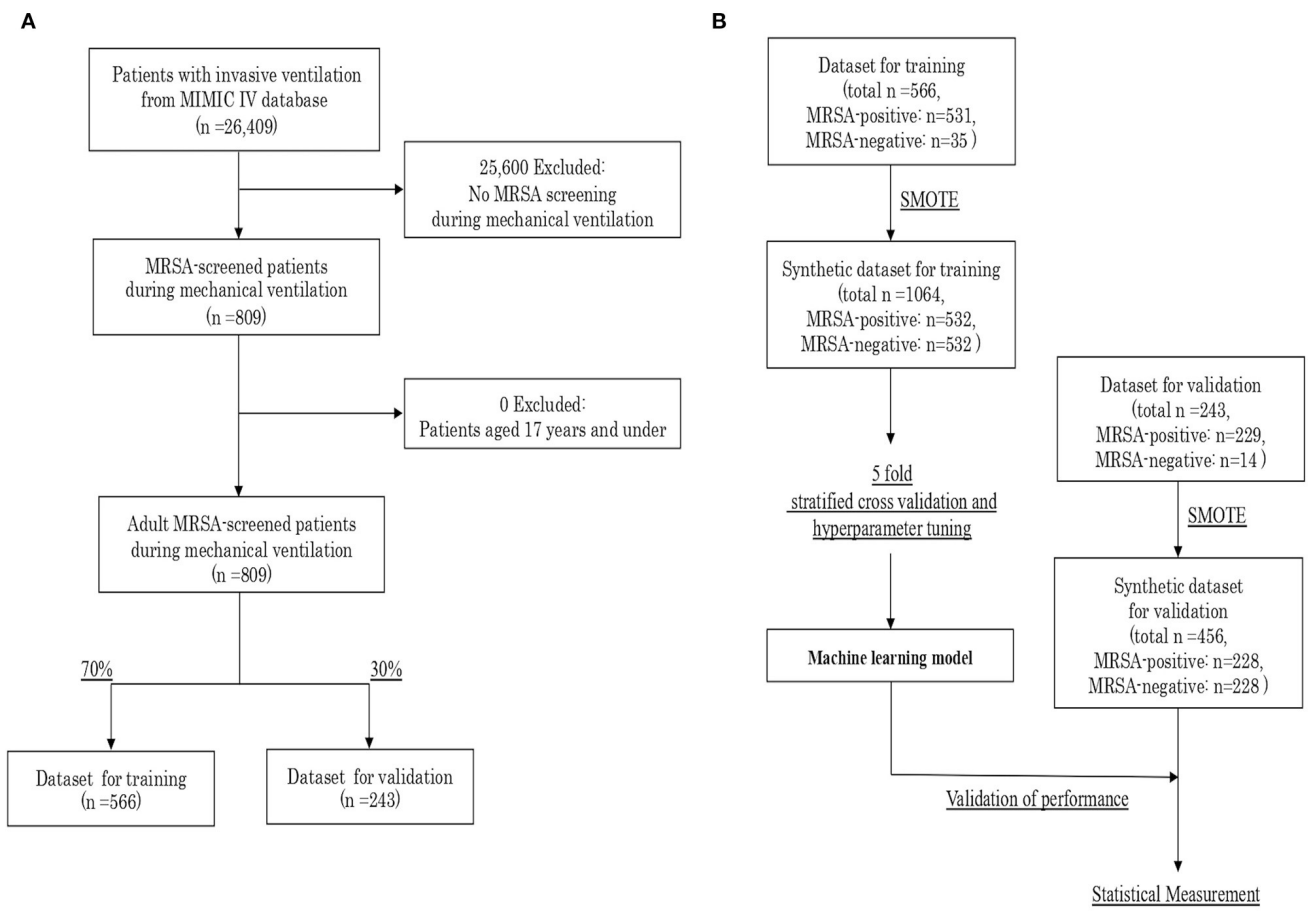
**(A)** Flow diagram of patient inclusion. **(B)** Procedure for creating the synthetic datasets and validating the machine learning model. MIMIC, Medical Information Mart for Intensive Care; MRSA, Methicillin-Resistant *Staphylococcus aureus*; SMOTE, Synthetic Minority Over-sampling Technique.

The study population were adult patients screened for MRSA during mechanical ventilation. The outcome was a MRSA-positive result on the screening test. A flow diagram of patient inclusion is shown in Figure 1A. Overall, 26,409 patients with invasive ventilation were identified from the MIMIC-IV database. Of these, 25,600 patients who were not screened for MRSA during the ventilated period were excluded. We meant to exclude also non-adult patients, aged 17 years and under, but no patients met this criterion. Thus, 809 adult patients MRSA-screened during mechanical ventilation were our included cohort. Finally, the subjects were divided into two groups by stratified random sampling with a 7:3 allocation ratio: a dataset for training (*n* = 566) and a dataset for validation (*n* = 243).

### Generation of Synthetic Datasets

The characteristics of the included cohort are shown in Supplemental Table 1. The outcome was highly imbalanced, with 93.9% of the patient classified as MRSA-positive by the screening test. As the imbalanced classification task is hard for predictive modeling due to the severely skewed class distribution and unequal misclassification costs, we created synthetic datasets with 50% of positive outcomes by synthetic minority over-sampling technique (SMOTE), independently for the training and validation datasets. SMOTE offers more related minority class samples to learn from, which leads to more coverage of the minority class (18). As the prevalence of MRSA screening test generally varies in individual countries and facilities, we set the outcome balance setting for the synthetic dataset at 50%, which is most balanced. We could generate a synthetic training dataset with a total of 1,064 samples, and a synthetic validation dataset with 456 samples (Figure 1B).

### Predictor Variables

In this study, 28 variables concerning pre-hospitalization information were selected as outcome predictors according to the availability of data from the MIMIC-IV and previous literature reviews on risk factors for MRSA (9, 12, 13, 19). These variables included age, sex, ICU locations, past medical history (diabetes mellitus, chronic obstructive pulmonary disease (COPD), chronic heart disease, cerebrovascular disease, peripheral vascular disease), Charlson comorbidity index, cellulitis, pressure ulcer, sequential organ failure assessment (SOFA) score at MRSA screening, acute physiology and chronic health evaluation (APACHE) III score on admission, admission from emergency department (ED), days spent at the hospital at the time of MRSA screening, days of ventilator use at MRSA screening, prior use of corticosteroids or antibiotics such as quinolone, macrolide, carbapenem, and interventional procedures (peripheral line, peripherally inserted central catheter (PICC) line, central venous catheter (CVC) line, pulmonary artery catheter (PAC) line, arterial line, urinary catheter, hemodialysis, and tracheostomy) before MRSA screening. ICU locations were handled as dummy variables, including medical intensive care unit (MICU), surgical intensive care unit (SICU), MICU/SICU, trauma surgical intensive care unit (TSICU), coronary care unit (CCU), cardiac vascular intensive care unit (CVICU), and other ICUs [neuro surgical intensive care unit (NSICU) or post anesthesia care unit (PACU)].

### Development and Validation of Machine-Learning Models

Using the synthetic training datasets, we trained and developed an XGBoost machine learning model as a classifier for outcome prediction. To avoid overfitting the model, we used five-fold stratified cross-validation. In addition, optimization of hyperparameters was performed to obtain the best performance in outcome prediction.

After the algorithm training process, the performance of the developed model was validated using the synthetic validation dataset. As statistical measures of performance, we calculated the area under the receiver operating characteristic (AUROC) curve, sensitivity, specificity, positive likelihood ratio, negative likelihood ratio, positive predictive value, negative predictive value, and accuracy. The process of machine learning and validation is described in Figure 1B. In addition, feature importance was computed as the normalized total reduction of the criterion brought by the feature, which is known as Gini importance.

### Statistical Analysis and Software Library for Machine Learning

Data were extracted from MIMIC-IV using structured query language (SQL) through Google Cloud's BigQuery platform. Statistical analyses of the characteristics of the cohorts were performed using SciPy (version 1.4.1) with Python (version 3.7.4, in Anaconda 2019.10). Age, as a continuous variable, was reported as mean and standard deviation. All categorical variables were reported as counts and percentages. The *t*-test was used to compare means between two samples. The chi-square test was used to compare frequencies. All tests were two-sided, and the significance level was set at 5% (*p* < 0.05). For model development, scikit-learn (version 0.21.3) with Python was employed.

## Results

### Characteristics of the Synthetic Datasets Used for Machine Learning

The characteristics of the synthetic datasets used for machine learning are shown in Table 1. The mean age in the synthetic training data was 66.6 ± 14.0 years, significantly older than that of the synthetic validation data (62.9 ± 15.6 years). A smaller fraction of patients admitted from ED or hospitalized in the CCU was present in the synthetic training data compared with the synthetic validation data (41.3% vs. 54.4% and 5.6% vs. 13.8%, respectively). Among procedures, peripheral line placement was performed significantly less frequently in the synthetic training data than in the synthetic validation data. The Charlson comorbidity index and the number of days of ventilator use at MRSA screening were also significantly different between the two datasets.

**Table 1:** Characteristics of the synthetic dataset used for machine learning.

**Variable**	**Synthetic training data (***n*** = 1,064)**	**Synthetic validation data (***n*** = 456)**	***P*** **-value**
Age (years)	66.6 [14.0]	62.9 [15.6]	<0.001
Gender (male)	528 (49.6%)	234 (51.3%)	0.73
**ICU location**
MICU	213 (20.0%)	73 (16.0%)	0.13
MICU/SICU	92 (8.6%)	33 (7.2%)	0.40
SICU	105 (9.9%)	47 (10.3%)	0.81
TSICU	76 (7.1%)	33 (7.2%)	0.95
CCU	60 (5.6%)	63 (13.8%)	<0.001
CVICU	137 (12.9%)	45 (9.9%)	0.14
Other (NSICU or PACU)	4 (0.4%)	3 (0.7%)	0.46
**Past medical history**
Diabetes Mellitus	162 (15.2%)	72 (15.8%)	0.81
COPD	15 (1.4%)	6 (1.3%)	0.89
Chronic heart disease	210 (19.7%)	77 (16.9%)	0.28
Cerebrovascular disease	88 (8.3%)	25 (5.5%)	0.08
Peripheral vascular disease	33 (3.1%)	14 (3.1%)	0.97
Charlson comorbidity index	5 (3–7)	6 (4–7)	0.01
Cellulitis	22 (2.1%)	9 (2.0%)	0.91
Pressure ulcer	381 (35.8%)	142 (31.1%)	0.22
SOFA score (at MRSA screening)	4 (2–6)	4 (2–7)	0.11
APACHE III score (on admission)	58 (41–78)	55(40–75)	0.26
Admision from ED	439 (41.3%)	248 (54.4%)	0.004
Length of hospital days (at MRSA screening)	3.0 [4.5]	2.9 [4.0]	0.39
Length of ventilator days (at MRSA screening)	1.9 [2.9]	1.9 [2.6]	0.03
**Prior antibitics use (before MRSA screening)**
Quinolone	75 (7.0%)	30 (6.6%)	0.76
Macrolide	25 (2.3%)	9 (2.0%)	0.66
Carbapenem	24 (2.3%)	12 (2.6%)	0.67
Prior corticosteroids use (before MRSA screening)	11 (1.0%)	5 (1.1%)	0.91
**Procedures (before MRSA screening)**
Peripheral line	675 (63.4%)	348 (76.3%)	0.03
PICC line	62 (5.8%)	36 (7.9%)	0.16
CVC line	284 (26.7%)	96 (21.1%)	0.07
PAC line	51 (4.8%)	32 (7.0%)	0.10
Arterial line	293 (27.5%)	146 (32.0%)	0.19
Urinary catheter	144 (13.5%)	66 (14.5%)	0.67
Hemodialysis	109 (10.2%)	44 (9.6%)	0.75
Tracheostomy	8 (0.8%)	1 (0.2%)	0.22
**Outcome**
MRSA-positive on screening test	532 (50.0%)	228 (50.0%)	1.0

*All categorical variables are shown as n (%). A continuous variable (Age) is shown as mean [standard deviation]. ICU, Intensive Care Unit, MICU, Medical Intensive Care Unit, SICU, Surgical Intensive Care Unit, TSICU, Trauma Surgical Intensive Care Unit, CCU, Coronary Care Unit, CVICU, Cardiac Vascular Intensive Care Unit, NSICU, Neuro Surgical Intensive Care Unit, PACU, Post Anesthesia Care Unit, COPD:Chronic Obstructive Pulmonary Disease, SOFA, Sequential Organ Failure Assessment, MRSA, Methicillin-Resistant Staphylococcus aureus, APACHE, Acute Physiology And Chronic Health Evaluation, ED, Emergency Department, PICC, Peripherally Inserted Central Catheter, CVC, Central Venous Catheter, PAC, Pulmonary Artery Catheter*.

### Performance of the Machine Learning Model

**Figure 2:**
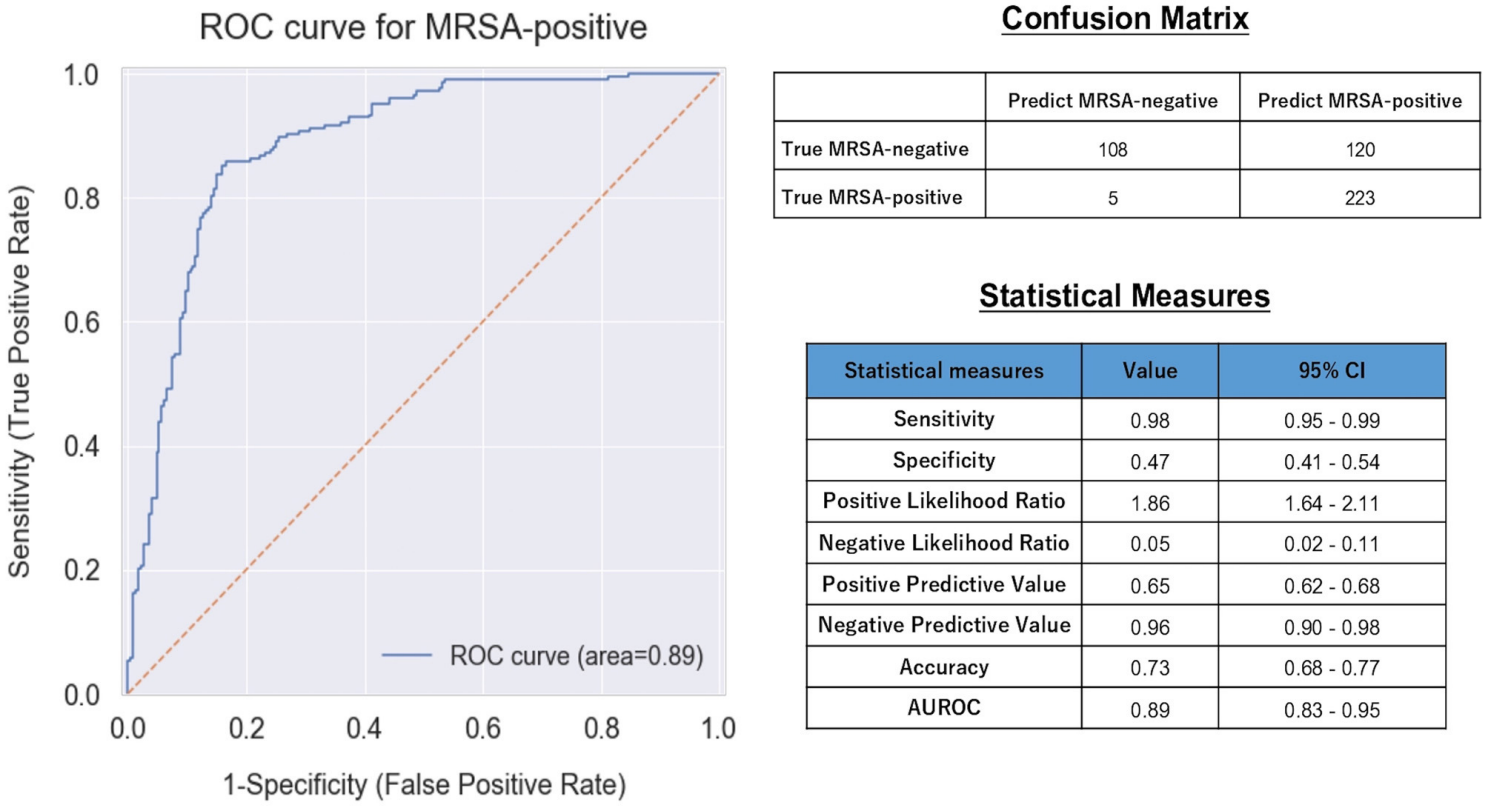
ROC curve, confusion matrix, and statistical measures of performance of the machine learning model. MRSA, Methicillin-Resistant *Staphylococcus aureus*; CI, Confidence interval; AUROC, Area Under the Receiver Operating Characteristic.

Figure 2 presents the ROC curve, AUROC value, confusion matrix, and statistical measures used to evaluate the performance of the machine learning model in the validation dataset. The ROC curve and its AUROC value showed good predictive ability of the model for MRSA-positivity in the screening test (AUROC: 0.89 [95% confidence interval (CI): 0.83–0.95]). Although the accuracy, specificity, and positive predictive value were relatively low (0.73 [CI: 0.68–0.77], 0.47 [CI: 0.41–0.54], and 0.65 [CI: 0.62–0.68], respectively), the model demonstrated a high sensitivity of 0.98 [CI: 0.95–0.99] and a high negative predictive value (0.96 [CI: 0.90–0.98]).

### Feature Importance

**Figure 3:**
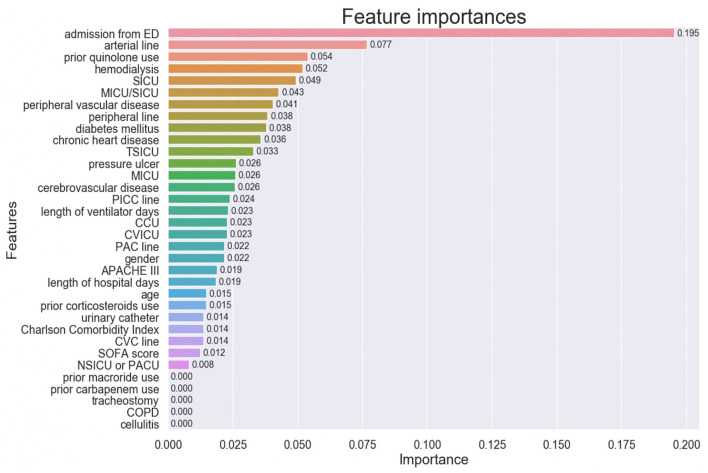
Feature importance of the model variables. ED, Emergency Department, SICU, Surgical Intensive Care Unit; MICU, Medical Intensive Care Unit; TSICU, Trauma Surgical Intensive Care Unit; PICC, Peripherally Inserted Central Catheter; CCU, Coronary Care Unit; CVICU, Cardiac Vascular Intensive Care Unit; PAC, Pulmonary Artery Catheter; APACHE, Acute Physiology and Chronic Health Evaluation; CVC, Central Venous Catheter; SOFA, Sequential Organ Failure Assessment; NSICU, Neuro Surgical Intensive Care Unit; PACU, Post Anesthesia Care Unit; COPD, Chronic Obstructive Pulmonary Disease.

The importance of the XGBoost model features is shown in Figure 3. Admission from ED was the most important variable in predicting MRSA-positivity in the screening test during mechanical ventilation. The five most important variables also included insertion of previous arterial lines, prior quinolone use, hemodialysis, and admission in the SICU, although they were far less important than admission from ED. Co-existing diseases such as peripheral vascular disease, diabetes mellitus, and chronic heart disease were also relatively important predictors. However, prior use of macrolide or carbapenem, tracheostomy, COPD, and cellulitis were of no importance in the predictive model.

## Discussion

In the current study, we undertook the development of a machine learning model to predict MRSA colonization during mechanical ventilation using the MIMIC-IV, a large open relational database containing data derived from the ICUs of a single center. As the extracted data were found to be highly imbalanced in terms of outcome, we created independent synthetic balanced datasets for training and validation by an oversampling technique. The machine learning-based model thus developed showed good performance in predicting MRSA screening positivity, with the reasonably high AUROC of 0.89.

Although previous large-scale studies have clarified the risk factors for MRSA colonization or infection, decision-making for the antimicrobial coverage of MRSA by critical care physician is still challenging. These risk factors are not specific, but rather common in critically ill patients, so that clinical practitioners cannot discriminate between MRSA-positive and negative patients without specimen testing. In this context, our current study supports the potential use of a machine learning model, which could be superior to human learning in predicting outcomes depending on complexly intertwined factors. Previously, Hartvigsen et al. reported the results of their challenge toward the prediction of MRSA-positive patients by machine learning models (20). They succeeded in developing a machine learning-based model which showed high predictive performance in the ICU patients. However, our study is novel in that we targeted the specific population of mechanically ventilated patients, who exhibit more severe conditions and are more susceptible to nosocomial infections, such as VAP, than those analyzed in the previous study. Broad-spectrum antibiotics including coverage for MRSA are frequently the initial choice by practitioners to treat these patients at high risk of death, thus the reliable prediction of MRSA colonization would more likely lead to a reduction of unnecessary antibiotics use.

Our prediction model showed low specificity and positive predictive value to predict MRSA colonization, indicating that the prediction of MRSA-positivity by the model does not guarantee positivity of the MRSA screening test. On the other hand, our model demonstrated high sensitivity and negative predictive value, implying that predicted MRSA negativity strongly supports the actual absence of MRSA colonization. The result of MRSA screening test does not promise the necessity of antibiotics coverage for MRSA. However, MRSA colonization is a high risk factor to develop MRSA infections in ICU patients (19). Therefore, acknowledgment of the presence of MRSA colonization as early as possible before the result of MRSA-screening test comes out might be helpful as one of the risk evaluations for MRSA infection, although other clinical conditions or examinations such as gram staining of the patients should be definitely considered to decide the use of antibiotics with coverage of MRSA. Real-time identification of the mechanically-ventilated patients who could potentially spread MRSA is also beneficial because this patient population requires medical practitioners to provide many contact opportunities for cares.

In this study, the model was created using 28 features that have been reported to be risk factors for MRSA colonization or infection in the previous literature, and that could be accurately extracted from the MIMIC-IV database. Among these features, admission from ED contributed the most to the prediction model. As the population of the study consisted of mechanically-ventilated patients, we presumed that patients admitted from ED might constitute an epidemiologically unique patient subgroup, distinct from those who were admitted in the ICU for the purpose of surgical operations. Patient admitted from ED could have more complex combinations of risk factors for MRSA colonization, including not only medical conditions or existing diseases, but also social backgrounds, such as transfer from residential care homes or homelessness (21, 22). In contrast, patient severity scores such as SOFA or APACHE III were less important predictors. It is reassuring that well known risk factors for MRSA, such as hemodialysis and arterial lines, were detected as important features for the prediction. The ICU location of admission (SICU or MICU/SICU) was also highly relevant to the prediction, although we cannot determine whether this was related to the transmission of MRSA itself or to differences in patient diagnosis in each ICU. As previously described elsewhere (23), the model identified prior use of quinolones as an important risk factors for MRSA, compared to carbapenem or macrolide. However, caution is required in the interpretation of the feature importance of each variable, because the percentage of positives for some of the assessed features was very low.

Our study has several limitations. First, we trained the model and validated it using synthetic datasets due to the severe class imbalance of the extracted datasets. The evaluation of the model on unrealistic data is the strongest limitation of the study, and could have led to an overly optimistic assessment of its performance, thus absolutely requiring external validation using real-world datasets with more balanced outcomes in the future. Second, we could not take into account how and why MRSA screening tests were performed in the included patients. In our dataset, the MRSA screening positivity rate was extremely high. Moreover, only 809 out of 26,409 patients were screened for MRSA during mechanical ventilation. These facts implied that clinicians might have decided to screen a patient for MRSA based on specific reasons such as clinically strong suspicion of MRSA positivity or MRSA screening protocol for the facility. The reasons physicians in the facility consider selecting patients for screening can also overlap with the predictors used to develop the model. These might have caused bias. Third, we could not include well-known risk factors for MRSA colonization such as pre-existing cancer, HIV infection, and intravenous drug use as predictive features, due to the insufficient information available from the dataset. Hence, the model is amenable to further improvements in performance. Finally, the model might not have worldwide generalizability because it was trained on a dataset derived from a single center, while the epidemiology of antimicrobial resistance differs among countries, hospitals and ethnicities (24, 25). It might be preferable to develop and use microbiome prediction models specific for each region or hospital.

## Conclusions

In conclusion, we were able to develop a machine learning model to predict positive screening for MRSA during mechanical ventilation using a synthetically augmented dataset from single center/MIMIC-IV database. Although external validation using more balanced, real-world datasets is required, the result of the current study demonstrated the possibility of early detection of MRSA in mechanically-ventilated patients by a machine learning approach, which might lead to optimized antibiotic selection by clinicians.

## Data Availability Statement

The raw data supporting the conclusions of this article will be made available by the authors, without undue reservation.

## Ethics Statement

The studies involving human participants were reviewed and approved by the Institutional Review Boards of the Beth Israel Deaconess Medical Center (BIDMC) and the Massachusetts Institute of Technology (MIT). Written informed consent for participation was not required for this study in accordance with the national legislation and the institutional requirements.

## Author Contributions

YH, JB, SM, MS, and PO: extracted data and conducted data cleaning. YH, KS, MS, and PO: analyzed the data. YH, KS, YO, and AT: interpreted the data. YH drafted the manuscript. All authors reviewed and discussed the manuscript. All authors read and approved the final manuscript. All authors jointly conceived of and designated this study.

## Funding

This research was supported by JSPS KAKENHI Grant Number 19H03764.

## Conflict of Interest

JB and SM were employed by the company Dowell Co., Ltd. MS was employed by the company DeerWalk Japan. The remaining authors declare that the research was conducted in the absence of any commercial or financial relationships that could be construed as a potential conflict of interest.

## Publisher's Note

All claims expressed in this article are solely those of the authors and do not necessarily represent those of their affiliated organizations, or those of the publisher, the editors and the reviewers. Any product that may be evaluated in this article, or claim that may be made by its manufacturer, is not guaranteed or endorsed by the publisher.

## Abbreviations

VAP: Ventilator-Associated Pneumonia
ICU: Intensive Care Unit
MRSA: Methicillin-Resistant *Staphylococcus aureus*
MIMIC: Medical Information Mart for Intensive Care
COPD: Chronic Obstructive Pulmonary Disease
SOFA: Sequential Organ Failure Assessment
APACHE: Acute Physiology and Chronic Health Evaluation
ED: Emergency Department
SICU: Surgical Intensive Care Unit
TSICU: Trauma Surgical Intensive Care Unit
CCU: Coronary Care Unit
AUROC: Area Under the Receiver Operating Characteristic
CI: Confidence Interval.
