# Supplementary material for: Machine Learning Approach to Predict Positive Screening of Methicillin-Resistant Staphylococcus aureus During Mechanical Ventilation Using Synthetic Dataset From MIMIC-IV Database
Source: Front Med (Lausanne). 2021 Nov 16;8:694520. doi: 10.3389/fmed.2021.694520 (PMC8635043; doi:10.3389/fmed.2021.694520)
Supplement: Supplementary file 1 [file Table_1.DOCX]

| Variable | All  (n=809) | Training data  (n=566) | Test data  (n=243) | P value |
| --- | --- | --- | --- | --- |
| Age (years) | 67.1 [16.0] | 67.4 [15.8] | 66.5 [16.6] | 0.48 |
| Gender (male) | 468 (57.8%) | 333 (58.8%) | 135 (55.6%) | 0.65 |
| **ICU location** |  |  |  |  |
| MICU | 237 (29.3%) | 168 (29.7%) | 69 (28.4%) | 0.78 |
| MICU/SICU | 119 (14.7%) | 86 (15.2%) | 33 (13.6%) | 0.60 |
| SICU | 142 (17.6%) | 104 (18.4%) | 38 (15.6%) | 0.43 |
| TSICU | 109 (13.5%) | 76 (13.4%) | 33 (13.6%) | 0.95 |
| CCU | 91 (11.2%) | 53 (9.4%) | 38 (15.6%) | 0.02 |
| CVICU | 104 (12.9%) | 75 (13.3%) | 29 (11.9%) | 0.65 |
| Other (NSICU or PACU) | 7 (0.9%) | 4 (0.7%) | 3 (1.2%) | 0.46 |
| **Past medical history** |  |  |  |  |
| Diabetes Mellitus | 196 (24.2%) | 145 (25.6%) | 51 (21.0%) | 0.27 |
| COPD | 21 (2.6%) | 15 (2.7%) | 6 (2.5%) | 0.88 |
| Chronic heart disease | 260 (32.1%) | 188 (33.2%) | 72 (29.6%) | 0.47 |
| Cerebrovascular disease | 106 (13.1%) | 81 (14.3%) | 25 (10.3%) | 0.17 |
| Peripheral vascular disease | 47 (5.8%) | 33 (5.8%) | 14 (5.8%) | 0.97 |
| Charlson comorbidity index | 5 (4-7) | 5 (4-7) | 4 (2-6) | 0.36 |
| Cellulitis  Pressure ulcer  SOFA score (at MRSA screening) | 31 (3.8%)  347 (42.9%)  4 (2-6) | 22 (3.9%)  246 (43.5%)  4 (2-6) | 9 (3.7%)  101 (41.6%)  4 (2-6) | 0.90  0.75  0.38 |
| APACHE III score (on admission)  Admision from ED | 61 (43-81)  539 (66.6%) | 60 (43-80)  371 (65.5%) | 64(43-81)  168 (69.1%) | 0.35  0.66 |
| Length of hospital days (at MRSA screening) | 3.4 [5.7] | 3.4 [5.9] | 3.4 [5.1] | 0.42 |
| Length of ventilator days (at MRSA screening) | 2.0 [3.7] | 2.0 [3.9] | 2.0 [3.2] | 0.50 |
| **Prior antibitics use (before MRSA screening)** |  |  |  |  |
| quinolone | 105 (13.0%) | 75 (13.3%) | 30 (12.3%) | 0.76 |
| macrolide | 34 (4.2%) | 25 (4.4%) | 9 (3.7%) | 0.66 |
| carbapenem | 32 (4.0%) | 20 (3.5%) | 12 (4.9%) | 0.37 |
| Prior corticosteroids use (before MRSA screening) | 10 (1.2%) | 5 (0.9%) | 5 (2.1%) | 0.17 |
| **Procedures (before MRSA screening)** |  |  |  |  |
| Peripheral line | 603 (74.5%) | 414 (73.1%) | 189 (77.8%) | 0.60 |
| PICC line | 98 (12.1%) | 62 (11.0%) | 36 (14.8%) | 0.17 |
| CVC line | 311 (38.4%) | 216 (38.2%) | 95 (39.1%) | 0.87 |
| PAC line | 50 (6.2%) | 35 (6.2%) | 15 (6.2%) | 1.00 |
| Arterial line | 307 (37.9%) | 219 (38.7%) | 88 (36.2%) | 0.65 |
| Urinary catheter | 114 (14.1%) | 73 (12.9%) | 41 (16.9%) | 0.20 |
| Hemodialysis | 146 (18.0%) | 106 (18.7%) | 40 (16.5%) | 0.52 |
| Tracheostomy | 9 (1.1%) | 8 (1.4%) | 1 (0.4%) | 0.22 |
| **Outcome** |  |  |  |  |
| MRSA-positive on screening test | 760 (93.9%) | 531 (94.0%) | 229 (93.8%) | 0.97 |

**Supplemental Table 1: Characteristics of MRSA-screened patients with mechanical ventilation.**

All categorical variables are shown as n (%). A continuous variable (Age) is shown as mean [standard deviation]. Four missing data observed in SOFA score were imputed by median value. ICU: Intensive Care Unit, MICU: Medical Intensive Care Unit, SICU: Surgical Intensive Care Unit, TSICU: Trauma Surgical Intensive Care Unit, CCU: Coronary Care Unit, CVICU: Cardiac Vascular Intensive Care Unit, NSICU: Neuro Surgical Intensive Care Unit, PACU: Post Anesthesia Care Unit, COPD:Chronic Obstructive Pulmonary Disease, SOFA: Sequential Organ Failure Assessment, MRSA: Methicillin-Resistant Staphylococcus Aureus, APACHE: Acute Physiology And Chronic Health Evaluation, ED: Emergency Department, PICC: Peripherally Inserted Central Catheter, CVC: Central Venous Catheter, PAC: Pulmonary Artery Catheter.
